# Timing of antenatal care for adolescent and adult pregnant women in south-eastern Tanzania

**DOI:** 10.1186/1471-2393-12-16

**Published:** 2012-03-21

**Authors:** Karin Gross, Sandra Alba, Tracy R Glass, Joanna Armstrong Schellenberg, Brigit Obrist

**Affiliations:** 1 Swiss Tropical and Public Health Institute, Basel, Switzerland; 2 University of Basel, Basel, Switzerland; 3 London School of Hygiene and Tropical Medicine, London, UK; 4 University of Basel, Institute of Anthropology, Basel, Switzerland

## Abstract

**Background:**

Early and frequent antenatal care attendance during pregnancy is important to identify and mitigate risk factors in pregnancy and to encourage women to have a skilled attendant at childbirth. However, many pregnant women in sub-Saharan Africa start antenatal care attendance late, particularly adolescent pregnant women. Therefore they do not fully benefit from its preventive and curative services. This study assesses the timing of adult and adolescent pregnant women's first antenatal care visit and identifies factors influencing early and late attendance.

**Methods:**

The study was conducted in the Ulanga and Kilombero rural Demographic Surveillance area in south-eastern Tanzania in 2008. Qualitative exploratory studies informed the design of a structured questionnaire. A total of 440 women who attended antenatal care participated in exit interviews. Socio-demographic, social, perception- and service related factors were analysed for associations with timing of antenatal care initiation using regression analysis.

**Results:**

The majority of pregnant women initiated antenatal care attendance with an average of 5 gestational months. Belonging to the Sukuma ethnic group compared to other ethnic groups such as the Pogoro, Mhehe, Mgindo and others, perceived poor quality of care, late recognition of pregnancy and not being supported by the husband or partner were identified as factors associated with a later antenatal care enrolment (p < 0.05). Primiparity and previous experience of a miscarriage or stillbirth were associated with an earlier antenatal care attendance (p < 0.05). Adolescent pregnant women started antenatal care no later than adult pregnant women despite being more likely to be single.

**Conclusions:**

Factors including poor quality of care, lack of awareness about the health benefit of antenatal care, late recognition of pregnancy, and social and economic factors may influence timing of antenatal care. Community-based interventions are needed that involve men, and need to be combined with interventions that target improving the quality, content and outreach of antenatal care services to enhance early antenatal care enrolment among pregnant women.

## Background

Maternal death has declined substantially worldwide except in Sub-Saharan Africa [1]. Of the 21 countries with the highest maternal mortality 15 are in sub-Saharan Africa, including Tanzania [1]. In 2010, pregnancy and childbirth-related complications led to an estimated 454 maternal deaths per 100'000 live births in Tanzania [2]. Most of these complications occur unpredictably during labour, delivery and the immediate postpartum period [3]. Deaths could be averted with prompt and adequate diagnosis and care [4]. However, 49% of all women in Tanzania still deliver at home without any skilled attendant [2]. Moreover, according to the definition of the World Health Organisation (WHO) [5] a quarter of all women in Tanzania begin childbearing as adolescents before reaching the age of 20 years [2]. An estimated 70'000 adolescent mothers die each year worldwide because their bodies are not yet physically ready for motherhood and due to social disadvantages [6,7]. Pregnancy and childbirth thus constitutes the number one killer among 15-19 year old girls worldwide [5].

Several studies have shown that women who started antenatal care (ANC) attendance early and attended frequently were more likely to be assisted during delivery by a skilled attendant compared to those who initiated ANC late and attended only few visits [8-11]. Although ANC might not have the potential to predict and avert obstetric emergencies during pregnancy and childbirth, it exposes women to health education on risk factors and encourages them to deliver with a skilled attendant or in a health facility. Recent studies have suggested that women who knew about risk factors were more likely to utilize health facilities for delivery than those without knowledge [10,12]. Moreover, ANC provides the opportunity to detect and treat anomalies of pregnancy and to deliver preventive health services such as immunization against tetanus, prophylactic treatment of malaria and worms, and HIV testing and counselling (leading to Preventing Mother to Child Transmission of HIV, PMTCT) [13]. To fully benefit from these interventions, it is important that women start ANC early on in their pregnancy. The revised Focused Antenatal Care (FANC) model of WHO [13] as well as the Tanzanian FANC guidelines [14] recommend at least four ANC visits for uncomplicated pregnancies with the first visit starting before 16 weeks of gestation [13]. However, an analysis of Demographic and Health Surveys (DHS) from 45 developing countries showed that women in sub-Saharan Africa start antenatal care considerably later than women from other regions [8]. Similarly, other studies reported late ANC enrolment after more than five months of gestation in sub-Saharan African countries [15-18], including Tanzania [2,10,19,20]. A comparative analysis of the use of maternal health services in sub-Saharan Africa showed that adolescent mothers initiated ANC attendance even later and had poorer maternal health care than adult mothers [21].

Quantitative studies on timing of ANC attendance from developing countries have been able to shed light on the influence of socio-demographic factors. Although there is mixed evidence, late booking of antenatal care has repeatedly been associated with young age [21-24], premarital status [21,23] unwanted pregnancies [16,23,25], high parity [16,21,23,26,27], lack of formal education [21-23,27], low socio-economic status (SES) [16,23] and ethnicity [16,27]. Less is known about the influence of social and cultural determinants on prenatal care use among adult and adolescent pregnant women [28]. Qualitative as well as quantitative studies have stressed the influence of social support from family members [24,29-31]. A study from Nepal for example reported the important role of mothers-in-law in deciding about ANC use of their pregnant daughters-in-law [30]. Studies from Uganda showed that adolescents were more likely to experience violence from parents, to be rejected by their partner, expelled from school, and to be stigmatized [29,32], and therefore to hide their pregnancy [32]. Late recognition of and uncertainty about the pregnancy [33-35], as well as cultural beliefs and practices around pregnancy [34-38], have been reported to influence women's timing of ANC attendance. Ethnographic studies from Mozambique and southern Tanzania illustrated for example that women at an early stage of pregnancy delayed ANC initiation purposely in order to protect the unborn from witchcraft and sorcery attacks of jealous neighbours and kin [36,37]. Other studies showed that women's ANC attendance is mediated by their experiences and the quality of care at earlier ANC visits [39,40]. These studies clearly indicate that beyond demographic and socio-economic factors, social and cultural factors as well as individual perception of pregnancy and care impact women's timing of ANC enrolment. Unfortunately, data are often not disaggregated by age, thus hiding particular vulnerabilities and issues [6].

Exploratory studies carried out in the study area in 2007 as a preparation for this study confirmed several of the factors stressed in the literature. In semi-structured interviews [41], health workers reported that women, and in particular women from the ethnic group of the Sukuma - semi-nomadic pastoralists who started to migrate into the region in the 1980s [42] - initiated ANC attendance late and underutilized ANC due to lack of education and living in distant settlements. Data collected between 2007 and 2009 from the Health Management Information System (HMIS) of the health facilities within the study area [41] indicated that the proportion of pregnant women who initiated ANC attendance after the fifth month of gestation rose from 53% to 56% between 2006 and 2008. Over this period, 18% of all ANC attendees were 19 years old or less. In an in-depth study with a small sample of recent adult and adolescent mothers (Gross 2007, unpublished data), adolescent women were found to visit the ANC clinic later and less frequently than adult women. Moreover, adolescent mothers differed from adult mothers in several ways: most of them were in their first pregnancy which was unplanned and prior to marriage, they still lived at their parents' home and they did not get any social or economic support from their partner or the child's father.

Based on the insights from the literature review and the exploratory studies, three main research questions arose that are addressed in this paper: First, do pregnant women - and in particular adolescent pregnant women - start ANC attendance late? Second, what factors are associated with early or late ANC attendance? And finally, do adolescent pregnant women differ from adult pregnant women in terms of social and economic support?

## Methods

### Study setting

Data collection took place in the Kilombero and Ulanga rural Demographic Surveillance System (DSS) site in south-eastern Tanzania between June and October 2008. The area consists of 25 villages and has been extensively described elsewhere [41,43-46]. At the time of study, the area encompassed an estimated population of nearly 94,000 [47] and was served by a total of 13 first and second-level health facilities. Out of these, 12 (ten public health facilities and two faith-based) facilities provided ANC services on a weekly or daily basis from Monday to Friday. Two district hospitals outside the study area serve as referral centres for complicated cases. The local health system runs a cost-sharing scheme from which pregnant women and children under five years of age are exempted. Besides the biomedical system, traditional birth attendants and traditional healers provide alternative sources of prenatal and delivery care in the area [47].

### Sample size and sampling procedures

A total of 440 pregnant women visiting an ANC clinic were recruited for a cross-sectional study using exit interviews. Ten facilities (nine government facilities and one faith based facility, five in the Kilombero district and five in the Ulanga district) were selected and visited for one day once per month. Since accessibility to health services in this rural context is constrained by seasonal conditions such as weather, agricultural work or availability of money, the exit interviews were conducted over several months. Two other health facilities in the study area (one government facility and one faith based facility) were not included in the study due to low levels of ANC attendance. On average, 12 randomly selected pregnant women were interviewed per visit (min-max: 1-21) adding up to a total of 43 pregnant women interviewed per health facility (min-max: 28-79). No formal sample size calculation was performed.

### Data collection

Two trained local female field workers interviewed the women. A questionnaire was used including closed and some open ended questions Additional file 1. The design of the questionnaire was informed by insights from the exploratory studies as well as findings from the literature. Questions focused on a) socio-demographic characteristics, b) women's knowledge about ANC services, perceived quality of care and motivation to attend the ANC clinics, c) social and cultural factors, and d) ANC service utilization. Information on perceived quality of care and health worker attitudes was only obtained from pregnant women who had attended an ANC clinic before the day of interview. Additionally, data on the number and timing of ANC visits received were copied from the ANC cards of all women. The questionnaire was designed in English, translated into Swahili, back-translated and pre-tested outside the study area.

### Data analysis

Data from the exit interviews were double-entered using Microsoft Access, validated with EpiInfo version 3.3.2 (EpiInfo Association, Denmark) and analysed in Stata 10 (StataCorp, College Station, Texas, USA).

To answer the first research question - whether pregnant women attended late - the mean gestational age of the foetus at the first visit was calculated and compared to national guidelines. For the comparison of means, t-tests were used. For the second research question - what factors were associated with an earlier or later ANC attendance - univariate and multivariate linear regression was used. All pre-specified variables with at least a 5% response in a category were included in the models. Two multivariate regression models were run. The first one included all variables. Since the sample size was substantially decreased by the high number of missing values for the variables on perceived quality of care and perceived health worker (N = 289), a second regression model excluding the two variables served as a sensitivity analysis (N = 372). The gestational age of the foetus in months at the first ANC visit served as the outcome measure. Data on the gestational age of the foetus was collected in gestational weeks from the ANC cards but had to be transformed into gestational months (assuming one month to encompass four weeks) to get a normal distribution needed for the regression analysis. The regression assessed associations between the women's timing of ANC initiation and socio-demographic, social, perception- and service related variables. Associations between variables were assessed with logistic regression models. To answer the third research question - whether adolescent pregnant women differed from adult pregnant women in terms of social and economic support - logistic regression models were fitted to understand associations between adolescent-hood and social and economic support received during pregnancy.

### Ethical considerations

The study was conducted within the frame of the ACCESS Programme which was cleared by the National Institution for Medical Research of Tanzania (NIMR/HQ/R.8c/Vol. I/66) [48]. Approval was further provided by the review boards of the Swiss Tropical and Public Health Institute (SwissTPH) and the Ifakara Health Institute (IHI). The study was authorized by the district coordinators of Reproductive-and-Child-Health (RCH) and the health facility staff granted permission to conduct the study at their facilities. All study participants provided oral informed consent after having been explained the purpose of the study and informed of their right to withdraw from the study at any time.

## Results

### Study population

Out of the 440 pregnant women who participated in the cross-sectional study, 35 women were excluded - 10 women because their ANC cards did not contain information on gestational age and 25 because information on their age was missing. The final sample consisted of a total of 405 participants, including 61 (15%) adolescents aged 19 years or younger. Table 1 summarizes the characteristics by age groups. The median age of all respondents was 25 years (Inter-quartile range (IQR) = 21-31) and 18 years (IQR = 17-19) among adolescents. Of all women interviewed, 20% were in their first pregnancy (primiparity); the median number of pregnancies was 3 (IQR = 2-5, including the current pregnancy). Among adolescent women, 79% were pregnant for the first time, and the median number of pregnancies was 1 (IQR = 1-1). A quarter of women (25%) reported a history of miscarriage or stillbirth. Most women (88%) were married or lived in a consensual union and 55% had completed 7 years of primary school. Women belonged to a wide mix of ethnic groups with the Pogoro (19%), Sukuma (17%), Mhehe (10%) and Mgindo (9%) being the most common.

**Table 1:** Characteristics of the sample

	Total sample			Sample of adult women			Sample of adolescent women		
**Categories**	**N**	**n**	**%**	**N**	**n**	**%**	**N**	**n**	**%**

**Socio-demographic characteristics**									
Age	405			344			61		
< 20 years		61	15					61	100
20-34 years		288	71		288	84			
35-49 years		56	14		56	16			
Parity	405			344			61		
1		82	20		34	10		48	79
2-4		218	54		205	60		13	21
5+		105	26		105	30			
Marital status	404			343			61		
Married/Consensual union		357	88		315	92		42	69
Single or separated		47	12		28	8		19	31
History of abort/stillbirth	402			342			60		
Abort/stillbirth		99	25		95	28		4	7
No abort/stillbirth		303	75		247	72		56	93
Education level	402			341			61		
No or incomplete education		180	45		156	46		24	39
Primary +		222	55		185	54		37	61
Ethnicity	405			344			61		
Sukuma		68	17		55	16		13	21
Other ethnic group		337	83		289	84		48	79

**ANC knowledge and perception**									
Perceived ANC starting time	383			327			56		
Within first 3 months		256	67		222	68		34	61
After first 3 months		127	33		105	32		22	39
Knowledge of services	405			344			61		
Low		314	78		260	76		54	88
High		91	22		84	24		7	12
Perceived service quality	319			266			53		
Good		292	91		241	91		51	96
Bad		27	9		25	9		2	4
Perceived health worker attitudes	310			258			52		
Good		291	94		239	93		52	100
Bad		19	6		19	7			
Traditional medicine use	402			341			61		
Yes		37	9		31	9		6	10
No		365	91		310	91		55	90

**Pregnancy perception**									
Early recognition of pregnancy	404			343			61		
Yes		284	70		240	70		44	72
No		120	30		103	30		17	28
Waiting for quickening	403			342			61		
Yes		108	27		94	27		14	23
No		295	73		248	73		47	77

**Social and economic support**									
Money	404			343			61		
Yes		256	63		221	64		35	57
No		148	37		122	36		26	43
Advice received to attend ANC	404						61		
Yes		175	43	343	129	38		46	75
No		229	57		214	62		15	25
Accompanied to clinic	405			344			61		
Yes		196	48		162	47		34	56
No		209	52		182	52		27	44
Supported by husband	405			344			61		
Yes		380	94		324	94		56	92
No		25	6		20	6		5	8

### Timing and reasons of ANC enrolment

Among the 405 pregnant women participating, only 29% initiated ANC attendance within the first four months of pregnancy as recommended by WHO [13] and the Tanzanian FANC guidelines [14]. Table 2 shows that overall pregnant women made their first ANC visit at a mean of 5.1 (SD = 1.2, range = 2-9). Adolescent pregnant women started slightly earlier with a mean of 5.0 months (SD = 1.2, range = 2-8). It is noteworthy that the 13 multiparous adolescents in the sample initiated ANC attendance considerably later with an average of 5.5 (SD = 1.20, t = 1.43; p = 0.157) gestational months (data not shown).

**Table 2:** Pregnant women's timing of ANC initiation

	Total sample			Sample of adult women			Sample of adolescent women		
**Categories**	**N**	**Mean**	**SD**	**N**	**Mean**	**SD**	**N**	**Mean**	**SD**

Mean gestational month at first ANC visit	404	5.1	1.2	344	5.1	1.2	61	5.0	1.2

When asked about their self-perceived timing of the first ANC visit, 56% of the participants said that they had made their first ANC visit late. Women who judged their first visit to be late attended ANC significantly later than women who perceived their first visit to be early (mean 5.5 gestational months vs. 4.7 months, t = 6.92, p < 0.001). Reasons given for late attendance were: not recognizing the pregnancy early (29%); poor accessibility due to distance, difficulties to cross rivers or poor road conditions (17%); not being able to come due to illness or other obligations such as travelling, caring for a sick person or agricultural work (14%); or negligence or apathy (13%). Women who said that they enrolled early in ANC did so in order to follow nurses' advise and because one is supposed to do so (37%); to know health status and prevent health problems (31%); out of fear that the consequences of non-compliance would lead to not being treated or being scolded by the health facility staff (16%); or to treat a health problem (15%).

#### Determinants of timing of ANC enrolment

##### Socio-demographic factors

Table 3 shows the results of the univariate and multivariate linear regression for all women. Being in the first pregnancy was strongly associated with an earlier ANC attendance. On average, primiparous women first visited ANC 0.87 month or three weeks earlier than multiparous women (p < 0.001). On the other hand we found no evidence of an association between timing of ANC attendance and adolescent age (p = 0.462). After adjusting for other factors, women who had a previous miscarriage or stillbirth attended 2 weeks earlier compared to women who had not experienced such an incident (p = 0.007). Although univariate analysis for all ethnic groups revealed some slight differences in the timing of first ANC attendance (results not shown), it was only statistically significant for members of the ethnic group of the Sukuma. Multivariate analysis revealed that compared to all other ethnic groups, being a member of the ethnic group of the Sukuma had a strong delaying effect on ANC initiation of three weeks (p < 0.001). There was no evidence that education (p = 0.987) or marital status (p = 0.532) were associated with an earlier or later timing of ANC attendance.

**Table 3:** Estimated effect of socio-demographic, social and perception- and service related factors on timing of pregnant women's first ANC visit in months

	Univariate			Multivariate (N = 289)		
	**Coeff^b^**	**(95% CI)**	**p-value**	**Coeff^b^**	**(95% CI)**	**p-value**

**Maternal factors**						
Parity (N = 405)						
Primi	-0.47	(-0.76, -0.18)	**0.002**	-0.87	(-1.32 -0.42)	**< 0.001**
Multi^a^						
Adolescent/adult (N = 405)						
Adolescent	-0.14	(-0.48, 0.19)	0.406	0.18	(-0.30, 0.65)	0.462
Adult^a^						
Marital status (N = 404)						
Married/Consensual union^a^						
Single	0.01	(-0.36, 0.39)	0.947	0.15	(-0.33, 0.63)	0.532
History of abort/stillbirth (N = 402)						
No abort ^a^						
One or more abort	-0.23	(-0.51, 0.05)	0.107	-0.46	(-0.79, -0.13)	**0.007**
Education level (N = 405)						
No or incomplete formal education	0.03	(-0.21, 0.27)	0.811	0.00	(-0.29, 0.29)	0.987
Primary + ^a^						
Ethnicity (N = 405)						
Sukuma	0.54	(0.23, 0.86)	**0.001**	0.82	(0.44, 1.19)	**< 0.001**
Other ethnic group^a^						

**ANC expectations, knowledge and perceived quality**						
Perceived ANC timing (N = 383)						
Within first 3 months ^a^						
After first 3 months	0.09	(-0.17, 0.36)	0.498	-0.05	(-0.35, 0.24)	0.727
Knowledge of services (N = 405)						
Low^a^						
High	0.30	(0.01, 0.59)	**0.042**	0.09	(-0.23, 0.41)	0.572
Perceived service quality (N = 319)						
Good^a^						
Bad	0.79	(0.30, 1.27)	**0.002**	0.78	(0.20, 1.36)	**0.009**
Perceived health worker attitudes(N = 310)						
Good^a^						
Bad	0.08	(-0.50, 0.67)	0.778	-0.57	(-1.21, 0.07)	0.082
Traditional medicine use (N = 402)						
Yes	-0.31	(-0.73, 0.11)	0.142	-0.07	(-0.55, 0.41)	0.759
No^a^						

**Pregnancy perception**						
Early recognition of pregnancy (N = 404)						
Yes^a^						
No	0.42	(0.16, 0.68)	**0.002**	0.47	(0.17, 0.77)	**0.002**
Waiting for quickening (N = 403)						
Yes	0.21	(-0.06, 0.48)	0.130	0.15	(-0.15, 0.45)	0.323
No^a^						

**Social and economic support**						
Money (N = 404)						
Yes^a^						
No	0.27	(0.02, 0.52)	**0.036**	0.28	(-0.02, 0.58)	0.064
Advised to attend ANC (N = 404)						
Yes	-0.11	(-0.35, 0.14)	0.386	-0.11	(-0.40, 0.18)	0.449
No^a^						
Accompanied to clinic (N = 405)						
Yes	-0.05	(-0.29, 0.20)	0.704	0.08	(-0.21, 0.37)	0.602
No^a^						
Supported by husband (N = 405)						
Yes^a^						
No	0.60	(0.10, 1.10)	**0.019**	0.69	(0.05, 1.33)	**0.035**

^a ^Reference category, ^b ^Estimated effect derived from linear regression on the timing of the first ANC visit expressed as gestational age in months (f.e. primiparous women started ANC on average 0.47 month earlier than multiparous women). The coefficients in the multivariate model are adjusted for all listed variables

##### Knowledge and perception of antenatal care

Neither women who said that ANC attendance should be initiated within the first three months of pregnancy (67%) nor those who had a good knowledge about ANC services (22%) were found to start ANC attendance earlier than the others. Although only few women criticized the quality of ANC services (9%), multivariate analysis showed that those who did so initiated ANC attendance an average of three weeks later compared to those who were satisfied by the quality (p = 0.009). Criticism was related to lack of services; being sent back home without receiving services due to the lack of sufficient staff; and having to purchase drugs, cards or diagnostic tests despite the national exemption policy that guarantees free health services for pregnant women. Surprisingly, perceived poor attitudes of health workers were associated with two weeks earlier attendance, although the effect was only marginally significant (p = 0.082). Few women sought treatment from sources other than ANC (9%) which was not associated with a late ANC initiation. Univariate logistic regression models revealed that women who reported that they had visited a traditional healer were more likely to have had a history of a reproductive loss (OR = 2.90, p = 0.003) or to belong to the Sukuma ethnic group (OR = 1.96, p = 0.09).

##### Knowledge and perception of pregnancy

Almost a third of the women interviewed (30%) said that they had not recognized early that they were pregnant, some of them because of continued bleeding or previous use of contraception. Multivariate regression showed that women's late perception of pregnancy was independently associated with a later ANC start of 2 weeks (p = 0.002). About a quarter of all women (27%) reported that they had waited for the foetus to move (quickening) before initiating ANC attendance. Although waiting for the quickening was not associated with a later ANC attendance (p = 0.323) in the first regression model, it became marginally associated (p = 0.088) with a later ANC start of one week in the sensitivity analysis that also included women attending the clinic for the first time in their life (data not shown).

##### Social and economic support

Table 3 illustrates the negative influence of lacking social and economic support on the timing of ANC initiation: In particular not possessing money in cash when attending the ANC clinic (p = 0.064) and not receiving support from the husband/partner (p = 0.035) were independently associated with a later ANC enrolment in the multivariate analysis for all women. Women who had no money in hand attended on average about 1 week later and women who felt not supported by their husband attended almost 3 weeks later than women who did receive such support. In the sensitivity analysis - including women who attended the ANC clinic for the first time in their life - not possessing money in cash when attending the ANC clinic became significantly associated with a later start of one week (p = 0.037) whereas there was not effect anymore for not receiving support from the husband/partner (p = 0.149, data not shown).

#### Social and economic support for adolescent women

Table 4 shows that adolescent pregnant women were less likely to be married or to live with their partner than adult pregnant women (p < 0.001). They were more likely to receive advice to attend the ANC (p < 0.001) and to have received this advice from their mother, or a close person they called 'mother', rather than from their partner compared to adult women (data not shown, OR = 9.83, p < 0.001).

**Table 4:** Estimated effect of being an adolescent on social/economic support

	Adolescent women	Adult women^a^			
	**n**	**n**	**OR ^b^**	**(95% CI)**	**p-value**

**Social and economic support**					
Marital status					
Married/Consensual Union^a^	42	315			
Single	19	28	5.09	(2.62-9.90)	**< 0.001**
Money					
Yes^a^	35	200			
No	26	122	1.35	(0.77-2.34)	0.293
Advice received to attend ANC					
Yes	46	129	5.09	(2.73-9.48)	**< 0.001**
No^a^	15	214			
Accompanied to clinic					
Yes	34	162	1.42	(0.82-2.45)	0.214
No^a^	27	182			
Supported by husband					
Yes^a^	56	324			
No	5	20	1.45	(0.52-4.01)	0.478

^a ^reference category, ^b ^Estimated effect of being an adolescent on social and economic support derived from logistic regression

## Discussion

This study showed that 71% of the pregnant women initiated ANC attendance after the recommended four months of pregnancy, at an average of 5.1 months (Table 2). This is consistent with the national average of 5 gestational months reported among facility users [19]. A DSS household survey conducted in the study area around the same time found a similar average of 5.02 gestational months at women's first ANC visit [47].

Adolescent pregnant women have been reported to most likely either not attend ANC or to attend late and infrequently [5,6,21,23,31,49] due to lack of knowledge, lack of power to take decisions, lack of money, or cultural factors including local concepts of illness [5]. Contrary to the findings of these studies and our exploratory studies, we found no evidence of delayed attendance in adolescents (Table 2 and 3). In line with an early study from the US that reported lower prenatal care utilization among adolescents in their second pregnancy [50] multiparous adolescents were found to start ANC attendance considerably later. Due to the study design of using exit interviews, we could only obtain information on women's timing of their first ANC visit and were unable to assess their overall utilization of ANC or even non-attendance. A study from Uganda comparing ANC attendance in adolescent and adult first time mothers found no difference in the timing of the first visit but a lower number of subsequent ANC visits in adolescents [29]. Similarly, Magadi et al. [21] found more variation by age with regard to frequency of ANC attendance than with timing. Little is known about adolescents ANC attendance in Tanzania, suggesting studies are needed to investigate their overall attendance.

Second, insights into factors influencing pregnant women's timing of ANC have been provided. Besides primiparity, having a history of a previous reproductive loss was found to be a strong predictor for an earlier ANC initiation in this study (Table 3). In accordance with other studies reporting that maternal care use varies across ethnic groups [16,47], the findings showed that the Sukuma ethnic group tended to have their first ANC visits later. Since Sukuma people live in very remote settlements of the study area, the effect is likely to be confounded by distance. GPS data is collected for each household within the DSS area, but unfortunately, it was not possible to merge this information with the demographic information collected during this study. Therefore neither data on distance between the homestead and the health facilities nor on women's socio-economic status were available for analysis. Some studies have reported an association between maternal secondary education and early timing of ANC initiation [21,51]. Contrary to these studies there was no evidence of such an effect in this study most presumably due to the overall low education level in the area, where few attend secondary school (see Table 1).

Women were well aware about their timing of ANC attendance, suggesting that confusion about the recommended starting time was not a problem. Few women (22%) could name more than four ANC services, but contrary to expectations, neither knowledge about correct ANC timing nor good knowledge of ANC services were associated with early ANC attendance (Table 3). Knowledge about available services might thus not imply that women are aware of the services' benefits. This matches with the surprisingly large number of women (53%) who indicated that they had attended ANC early because everyone does so, because of nurses' advice or because they feared the consequences of non-compliance with nurses' rules. In the exploratory study women indicated that their principal reason for attending the ANC clinic was to obtain an ANC card which was perceived as a necessary 'entry ticket' for services during delivery and illness rather than any conviction that ANC was good for their own or their child's health. The important pull factor of the ANC card has previously been reported by studies from Tanzania [20,52], South Africa [35], Malawi [34] and Uganda [40,53]. Attending ANC to obtain an ANC card thus might be one precautionary measure for women to conform to nurses' rules and to avoid harassment or informal payment requirements [53,54]. The extremely high rate of overall ANC attendance with 99.6% [47] and an average of 3.1 visits to ANC clinics over the course of their pregnancy (personal communication: M. Alexander) reported from the area, suggests that by creating informal rules nurses successfully force women to attend the ANC clinics, however not necessarily at an early point of time. This is of course no reason to excuse nor to foster negative attitudes on the side of the nurses towards their clients, but rather calls for better health education in health facilities, outreach services and in the community. Trained focal persons such as TBAs, religious leaders and other opinion leaders working as community volunteers in close collaboration with existing community structures and health services have been found to be effective promoters of obstetric care but also of early and frequent utilization of ANC in Southern Tanzania [55].

Perceived quality of care was generally high among the participants compared to a study from Kenya where almost a third of women complained about incomplete and inadequate services [31]. Considering that ANC services were of similarly low quality in the study area [56], women's high appraisal of the quality of ANC services rather reflects their low expectations of health care services. The fact that the interviewees were recruited at the health facility and interviewed in the proximity of the health facilities also potentially affected women's answers. Among those who were not satisfied with the services provided, perceived poor quality was, however, a strong predictor for late ANC attendance (Table 3). The findings indicate that quality of care, including patient-provider-relationship, plays a critical role in determining a woman's utilization of health care services, and needs to be improved but also better understood. In particular patient-provider-relationships should be further investigated through observational in-depth studies.

Late recognition of pregnancy was found to be a strong predictor of delayed ANC attendance in this study (Table 3). Similarly, late recognition of pregnancy and subsequent delay of ANC attendance has also been reported among South African women who received long acting hormonal contraceptives in the form of injections [33]. Although pregnancy tests seem to be available at drug shops in the study area at a price of between 500-1000 TSh (~0.30-0.60 USD) they are not widely used (personal communication: I. Mayumana). More than a quarter of participating women said they waited for the quickening before initiating ANC attendance. However, due to the limitation of quantitative methods to investigate topics that need more in-depth inquiry and trust for people to discuss them openly, this study was not able to explain whether women only waited to ensure pregnancy or also due to other reasons. Studies from Tanzania and other sub-Saharan countries have shown that late disclosure of the pregnancy due to local practices or beliefs such as witchcraft is common and has a negative influence on the timing of ANC attendance [34,36,37,55].

The study provides evidence for the negative influence of lacking social and financial support on women's timing of their first ANC visit and the key role of the husband or partner. The results legitimize the attempts of the Tanzanian Ministry of Health and Social Welfare to encourage greater male involvement in maternal health issues [57-59] in the sense that they are better informed about maternal health risks and live up to the expectations of support towards their children and their mothers. It is important, however, that this effort does not stop at policy level but reaches down to the health facility and community level. The community-based intervention conducted in Southern Tanzania found that in particular the equal inclusion of male community volunteers to promote obstetric care and early and frequent ANC use during home visits was an effective strategy to involve and inform men [55]. Supporting income generating activities for women such as revolving funds might be a suitable mean to reduce delay due to lack of economic means needed for ANC or other maternal health services particularly among women who lack support from their husband or partner.

Third, exploratory analysis comparing adolescent and adult pregnant women in terms of social and economic support during pregnancy confirmed that adolescent pregnant women were less likely to be married than adult pregnant women. These findings are in line with a study from Uganda reporting that adolescent first time mothers were more disadvantaged in terms of their likelihood to be rejected by partners [29]. On the other hand, in line with a study from Kenya [31], adolescents in this study were more likely to receive advice to attend the ANC clinic than adult women. However, this advice was mostly given by their mother than by their husband, partner or the child's father. These findings suggest that close family members rather than the husbands, partners and child fathers play an important role in supporting adolescent pregnant women. The fact that lacking support from the husband or partner showed no significant effect in the sensitivity analysis that included young women who visited the ANC clinic for the first time (data not shown) further supports this argument.

While the support of relatives seems to be sufficient for adolescents to initiate ANC around the same time as adult pregnant women, the consequences of disadvantages in terms of social and economic support on the overall ANC attendance and - even more importantly - for delivering with skilled attendance and postnatal care for themselves and their child needs to be further investigated.

## Conclusions

The majority of pregnant women delayed ANC attendance starting at an average of five months gestation. Adolescents had no greater delay in ANC initiation than adult pregnant women despite being more likely to be single. However, first ANC attendance at four months is recommended, so it is likely that some women missed important services offered during ANC such as preventive health measures, risk screening and health education.

This study found that many women rather attended due to norms and rituals than awareness about the health benefit of prenatal care; and that they delayed ANC initiation due to late perception of pregnancy, perceived bad quality of care and lack of social and economic support. These findings call for combined interventions at the community and health system level. Promotion of early and frequent ANC utilization through community-based interventions - involving also male community volunteers - could potentially be scaled up at low cost and adapted to local needs. Supporting income generating activities for women such as revolving funds might complement the approach in order to reduce delay due to lack of economic means needed for ANC or other maternal health services. At the same time, the quality of antenatal care services needs to be improved to attract women to use medical care throughout pregnancy, birth and the postpartum period; outreach services should be offered on a regular basis in order to bring services closer to women living in very distant settlements; and informal rules created by health workers in order to force women to attend the ANC clinic should be replaced with informing women about the benefits of maternal health services, but also the use of pregnancy tests.

## Competing interests

The authors declare that they have no competing interests.

## Authors' contributions

KG was responsible for the design and implementation of the study, carried out the data collection, the data management and analysis, and wrote the manuscript. SA assisted with data management, statistical analysis and contributed to the interpretation of the results and the discussion of the manuscript. TRG supported statistical analysis and commented on the manuscript. JS and BO supported the design and coordination of the study and contributed to the discussion of the manuscript. All authors have read and approved the final manuscript.

## Pre-publication history

The pre-publication history for this paper can be accessed here:

http://www.biomedcentral.com/1471-2393/12/16/prepub

## Supplementary Material

Additional file 1 **Questionnaire**. Click here for file
