# Supplementary material for: Timing of antenatal care for adolescent and adult pregnant women in south-eastern Tanzania
Source: BMC Pregnancy Childbirth. 2012 Mar 21;12:16. doi: 10.1186/1471-2393-12-16 (PMC3384460; doi:10.1186/1471-2393-12-16)
Supplement: Additional file 1 — Questionnaire. [file 1471-2393-12-16-S1.DOC]

| - **Greet the woman, ask her how she is doing.** - **Introduce yourself by name and organization.** - **Explain that you would like to get some information on ANC attendance.** - **Ask her for permission for the interview.**   **Information and invitation to participate in research:**  Our names are ......................., and we are interviewers from the Ifakara Health Research and Development Centre. We are working for the ACCESS Programme. We would like to learn about pregnant women’s opinion about the services offered in the ANC clinics and about their attendance. As you are experienced in these issues we would like to learn from your experience. The results of this research will help to improve the ANC services offered to pregnant women.  Therefore we would like to learn from your experiences. We can’t compensate you for the time and information you give us, but you, the pregnant women and mothers will hopefully be able to benefit from improved services at the health centres in the future. You will not loose time as we will make sure that you can return into the line again or that you will be attended directly after the interview.  We would greatly appreciate your co-operation in this research. The interview will only last a few minutes.  The information obtained from these conversations will be confidential, and is for research purposes only. Your name will not appear in any report that comes out of this study. Information from this research will be used for improving services only.  Do you have any questions? Do you agree to participate? Yes  No   - **Take her to a place where you can conduct the interview confidentially.** |
| --- |

| I. DSS INFORMATION |
| --- |

| **Date:** | **Day** | **Month** | **Year** | **Health Facility:** |  | **Interviewer code:** |  |
| --- | --- | --- | --- | --- | --- | --- | --- |

| 1. **Woman’s Name:** | **First** | **Last** |
| --- | --- | --- |

| 1. **Name of the household head** | **First** | **Last** |
| --- | --- | --- |

| 1. **Name of ten-cell leader:** | **First** | **Last** |
| --- | --- | --- |

| 1. **Home Village** |  | 1. **Kitongoji** |  |
| --- | --- | --- | --- |

| 1. **“When have you been born?”** | *🡪 fill in 88 if she doesn’t know* | [____]/[____]/[__­­­______] |
| --- | --- | --- |

**II. DEMOGRAPHIC DATA**

| 1. **“To which ethnic group do you belong?”** | | | |
| --- | --- | --- | --- |
| Choose only one answer | | | |
|  | 1 = Mdamba | 6 = Mbunga | [____] |
|  | 2 = Mpogoro | 7 = Mbena |
|  | 3 = Mhehe | 8 = Mngindo |
|  | 4 = Mskuma | 99 = other___________________________ |
|  | 5 = Mnyakyusa | ____________________________________ |

| 1. **“How many years have you been studying in school?”** | | | *Do not count the years that were not concluded* |  | **years** |
| --- | --- | --- | --- | --- | --- |
| **8.1** **“Did you go to secondary school?”** | | | | Choose only one answer | |
|  | 1 = Yes | 0 = No | |  | [____] |

**III. MOTIVATION TO ATTEND AND KNOWLEDGE ABOUT ANC SERVICES**

| 1. **„Why have you decided to attend the ANC clinic?“** | | 1 = Mentioned  2 = Mentioned with Question 9.1.  0 = Not mentioned | |
| --- | --- | --- | --- |
|  | |
|  | 1. To get the ANC card |  | [____] |
|  | 2. To know about the condition of the baby |  | [____] |
|  | 3. To prevent miscarriage |  | [____] |
|  | 4. To prevent problems |  | [____] |
|  | 5. To get treatment for a health problem |  | [____] |
|  | 6. To get services (IPTp, tetanus, etc.) |  | [____] |
|  | 7. To get the Hati Punguzo voucher |  | [____] |
|  | 8. Because we are told to go |  | [____] |
|  | 9. Because everyone goes |  | [____] |
|  | 10. I don’t know | *🡪 fill in 88* | [____] |
|  | 11. Other_________________________________________  ________________________________________________ | *🡪 fill in 99* | [____] |
| **9.1 „Is there any other reason?“** Continue to fill answers into the column above | | | |

| 1. **“What services should pregnant women receive if they attend the ANC clinic?”** | | 1 = Mentioned  2 = Mentioned with Question 10.1.  0 = Not mentioned | |
| --- | --- | --- | --- |
|  | 1. Measurements/Examinations |  | [____] |
|  | 2. Health education |  | [____] |
|  | 3. HIV/AIDS tests |  | [____] |
|  | 4. Blood tests |  | [____] |
|  | 5. Urine/stool tests |  | [____] |
|  | 6. Hati Punguzo Voucher |  | [____] |
|  | 7. SP/Malaria prevention |  | [____] |
|  | 8. Tetanus vaccine |  | [____] |
|  | 9. Folic/ferrous tablets |  | [____] |
|  | 10. Birth preparation plans |  | [____] |
|  | 11. I don’t know | *🡪 fill in 88* | [____] |
|  | 12. Other________________________________________  ________________________________________________ | *🡪 fill in 99* | [____] |
| **10.1 „Is there any other service that you should be given?“** Continue to fill answers into the column above*.* | | | |

| 1. **“What should you get as malaria prevention?”** | | 1 = Mentioned  2 = Mentioned with Question 11.1.  0 = Not mentioned | |
| --- | --- | --- | --- |
|  | Hati Punguzo voucher |  | [____] |
|  | A mosquito net |  | [____] |
|  | SP/Fansidar |  | [____] |
|  | Anti-malarial/Tablets |  | [____] |
|  | I don’t know | *🡪 fill in 88* | [____] |
|  | Other ___________________________________________  ________________________________________________ | *🡪 fill in 99* | [____] |
| **11.1. “Is there anything else you should get for malaria prevention?** Continue to fill answers into the column above | | | |

| 1. **“Why do they provide you with malaria prevention when you go to the ANC clinic?”** | | 1 = Mentioned  0 = Not mentioned | |
| --- | --- | --- | --- |
|  | Pregnant women are more at risk |  | [____] |
|  | To protect the child |  | [____] |
|  | To protect the mother |  | [____] |
|  | I don’t know | *🡪 fill in 88* | [____] |
|  | Other________________________________________ | *🡪 fill in 99* | [____] |

| 1. **“When are pregnant women supposed to start attending ANC services?”** |
| --- |
| **_________________________________________________________________________________________**  **_________________________________________________________________________________________**  **_________________________________________________________________________________________** |

| II. ACCESSIBILITY AND TRAVELING |
| --- |

| 1. **„Have you gone to any other ANC clinic during your current pregnancy?“** | | | | |
| --- | --- | --- | --- | --- |
| Choose only one answer | | | | |
|  | 1 = Yes **🡪 Q 14.1** | 0 = No **🡪 Q 15** |  | [____] |
| **14.1. „Why did you go to a different clinic?“**  **_________________________________________________________________________________________**  **_________________________________________________________________________________________**  _________________________________________________________________________________ | | | | |

| 1. **“How did you come to the ANC clinic?”**🡪 ***Probe*** 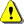 | | | | | |
| --- | --- | --- | --- | --- | --- |
| Choose only one answer | | | | | |
| 1 = By foot | 2 = Biking | 3 = Lift with bike | 4 = Car/Bus | 99 = other | [____] |

**IV. PERCEIVED QUALITY OF ANC SERVICES**

| 1. **„Have you ever attended ANC services before?“** | | | | |
| --- | --- | --- | --- | --- |
| Choose only one answer | | | | |
|  | 1 = Yes **🡪 Q.17** | 0 = No **🡪 Q. 20** |  | [____] |

| 1. **“How do you perceive the services that you get at this ANC clinic? Are they….”***🡪* ***probe*** 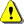 | | | | |
| --- | --- | --- | --- | --- |
| Choose only one answer | | | | |
|  | 1 = good | 2 = bad | 88 = I don’t know | [____] |
| **17.1. “Can you explain us please why do you think so?”**________________________________  ________________________________________________________________________________  ________________________________________________________________________________ | | | | |

| 1. **“How do you perceive the behavior of the health staff? Is their behavior …..?”***🡪* ***probe*** 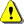 | | | | |
| --- | --- | --- | --- | --- |
| Choose only one answer | | | | |
|  | 1 = friendly | 2 = bad | 88 = I don’t know | [____] |
| **18.1. “How does she attend you?”**____________________________________________________  __________________________________________________________________________________________________________________________________________________________________ | | | | |

| 1. **“How do you consider the nurse’s competence? Is it …..?”***🡪* ***probe*** 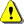 | | | | |
| --- | --- | --- | --- | --- |
| Choose only one answer | | | | |
|  | 1 = good | 2 = bad | 88 = I don’t know | [____] |

| V. POSSIBLE FACTORS FOR DELAY |
| --- |

| 1. **“According to your opinion, have you started early or late to attend the ANC services during this pregnancy”?** | | | | |
| --- | --- | --- | --- | --- |
| Choose only one answer | | | | |
|  | 1= early **🡪 Q. 20.1** | 0 =late **🡪 Q. 20.2** |  | [____] |
|  |  | | | |
| **20.1. „What was the reason that you started early?”**  **20.2. „What was the reason that you started late?”**  _________________________________________________________________________________  _________________________________________________________________________________  _________________________________________________________________________________  _________________________________________________________________________________ | | | | |

| 1. **“Did you realize early that you are pregnant?”** | | | | | | |
| --- | --- | --- | --- | --- | --- | --- |
| Choose only one answer | | | | | | |
|  | 1 = Yes **🡪 Q 21** | 0 = No **🡪 Q 21.1** | |  | | [____] |
|  | | | | | | |
| **21.1. „Why not?”** | | | 1 = Mentioned  0 = Not mentioned | | | |
|  | Because I thought it is another problem | |  | | [____] | |
|  | Because I still had menstrual bleedings | |  | | [____] | |
|  | Because I did not pay any attention | |  | | [____] | |
|  | Because I don’t have a regular menstrual bleeding | |  | | [____] | |
|  | Other_______________________________________ | | *🡪 fill in 99* | | [____] | |

| 1. **“Did you wait for the foetus to move before attending the ANC clinic?”** | | | | |
| --- | --- | --- | --- | --- |
| Choose only one answer | | | | |
|  | 1 = Yes **🡪 Q 22.1** | 0 = No **🡪 Q 23** |  | [____] |
| **22.1. “Why did you wait?**____________________________________________________________  __________________________________________________________________________________  __________________________________________________________________________________  __________________________________________________________________________________ | | | | |

| 1. **“Did you tell anyone that you are pregnant?”** | | | | |
| --- | --- | --- | --- | --- |
| Choose only one answer | | | | |
|  | 1 = Yes **🡪 Q 23.1** | 0 = No **🡪 Q 23.2** |  | [____] |
|  | | | | |

| **23.1 “Whom did you tell that you are pregnant?”** | | 1 = Mentioned  0 = Not mentioned | |
| --- | --- | --- | --- |
|  | Husband/partner/father of the child |  | [____] |
|  | Mother |  | [____] |
|  | Grandmother |  | [____] |
|  | Other family members |  | [____] |
|  | Friends |  | [____] |
|  | Neighbors |  | [____] |
|  | Other ___________________________________ | *🡪 fill in 99* | [____] |
| **23.2** 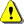 *If she did not tell anyone:* **“Why didn’t you tell anyone?”** _________________________  _________________________________________________________________________________________  _________________________________________________________________________________  _________________________________________________________________________________ | | | |
| 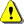*If she says that she did not tell anyone because she was afraid, also ask what was afraid of and write it down above.* | | | |

| 1. **„Did someone advise you to go to the ANC clinic?“** | | | | |
| --- | --- | --- | --- | --- |
| Chakua jibu moja tu | | | | |
|  | 1 = Ndiyo **🡪 Q 24.1** | 0 = Hapana |  | [____] |
| **24.1. “Who advised you to go?”**______________________________________________________  __________________________________________________________________________________ | | | | |

| 1. **„Did you come with someone else to the ANC?“** | | | | |
| --- | --- | --- | --- | --- |
| Choose only one answer | | | | |
|  | 1 = Yes | 0 = No |  | [____] |

| 1. **“Are you already married?”** [Demographic data] | | | | | |
| --- | --- | --- | --- | --- | --- |
| Choose only one answer | | | | | |
|  | 1 = Yes | | 0 = No |  | [____] |
| **26.1. “Are you currently living with your husband/partner?”**[Demographic data] | | | | |  |
| Choose only one answer | | | | | |
|  | | 1 = Yes | 0 = No |  | [____] |

| 1. **„Does your husband or partner supports you in any form going to the ANC clinic? F.e. did he advice you to come here, did he bring you, did he give you any money?“** | | | | |
| --- | --- | --- | --- | --- |
| Choose only one answer | | | | |
|  | 1 = Yes | 0 = No |  | [____] |

| 1. **“Do you right now have any money with you if you are asked to pay for drugs or services?”** | | | | |
| --- | --- | --- | --- | --- |
| Choose only one answer | | | | |
|  | 1 = Yes **🡪 Q 27.1** | 0 = No **🡪 Q 28** |  | [____] |
|  | | | | |

| **28.1. „Is it your money or from whom did you get it?”** | | 1 = Mentioned  0 = Not mentioned | |
| --- | --- | --- | --- |
|  | It’s my own money |  | [____] |
|  | From the husband/partner/father of the child |  | [____] |
|  | From other family members |  | [____] |
|  | Neighbor |  | [____] |
|  | Other______________________________________ | *🡪 fill in 99* | [____] |

| 1. **“Have you sought any care from one of the following sources of antenatal care during this pregnancy?”**   *🡪* ***probe*** 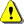 | | 1 = Mentioned  0 = Not mentioned | |
| --- | --- | --- | --- |
|  | From a TBA |  | [____] |
|  | From Mobiles/outreaches |  | [____] |
|  | Traditional healer |  | [____] |
|  | Drug shop |  | [____] |
|  | Other _______________________________________ | *🡪 fill in 99* | [____] |

| **VI. INFORMATION ON THE CURRENT PREGNANCY AND ANC ATTENDANCE** |
| --- |

| 1. **“How far are you in your pregnancy?”**   ____________________________________________________________________________________________________________________________________________________________________ |
| --- |

| 1. **“When do you expect to deliver?”**   ________________________________________________________________________________  ________________________________________________________________________________ |
| --- |

| 1. **“How many times have you been pregnant incl. this pregnancy?”** | [____] |
| --- | --- |

| 1. **“Have you ever experienced any miscarriage or stillbirth during another pregnancy?”** | | | | |
| --- | --- | --- | --- | --- |
| Choose only one answer | | | | |
|  | 1 = Yes | 0 = No |  | [____] |

| V. INFORMATION FROM ANC CARD |
| --- |

| 1. **“Is this the first time for you to attend the ANC clinic during this pregnancy?”** | | | | |
| --- | --- | --- | --- | --- |
| Choose only one answer | | | | |
|  | 0 = No **🡪 34.1** | 1 = Yes **🡪 35.2** |  | [____] |
|  | | | | |
| **34.1 “How many times have you attended the ANC clinic during this pregnancy, incl. today”?**  88 = I don’t know | | | | [____] |
|  | | | | |
| **35.1** 🗣 **For pregnant women who are attending for the second or more times:**  **“Asante sana. We have finished with our interview, but there is some information we would like to copy from your ANC card. Could you give us your ANC card, please?”**  **🡪 Continue with question 36** | | | | |

| **35.2.** 🗣 **For pregnant women attending for the first time:**  ***“*Asante sana. We have finished with our interview, but there is some information we would like to get after you have been attended at the clinic. Could you please come to see us again before you leave? It is very important that you come again.**  **🡪 Continue with question 38** |
| --- |

| 1. **“How often have you been given SP for malaria prevention during this pregnancy”?**   88 = I don’t know | [____] |
| --- | --- |

| 1. **“Did you receive a Hati Punguzo Voucher during this pregnancy”?** | | | | |
| --- | --- | --- | --- | --- |
| Choose only one answer | | | | |
|  | 1 = Yes | 0 = No |  | [____] |

| 1. **Data from the ANC card:** 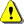*Please copy all the information from the indicated fields.* |
| --- |

| **KADI HII HAIUZWI**  **Jamhuri ya Muungano wa Tanzania**  **Wizara ya Afya na Ustawi wa Jamii**  **KADI YA KLINIKI YA WAJA WAZITO**  **Jaza au weka (√) panapohusika** | | |
| --- | --- | --- |
| JINA LA KLINIKI | NAMBA YA UANDIKISHAJI |  |
|  | NAMBA YA HATI PUNGUZO |  |

| **REKODI YA MAHUDHURIO**  CHUNGUZA VYOTE KILA HUDHURIO MPELEKE KITUO CHA AFYA /  HOSPITAL IWAPO KIWANGO KINAZIDI AU KUPUNGUA  ILIYO KWENYE MABANO  **Mimba isiyo na matatizo mama anahitaji mahudhurio 4: chini ya wiki 16, kati ya wiki 20-24, 28-32, 36-40*.** | | | | | | |
| --- | --- | --- | --- | --- | --- | --- |
| **TEREHEYA HUDHURIO** |  |  |  |  |  |  |
| UZITO (Kilo) |  |  |  |  |  |  |
| BLOOD PRESSURE (140/90mmHg) |  |  |  |  |  |  |
| ALBUMIN KWENYE MKOJO (+) |  |  |  |  |  |  |
| DAMU/Hb (8.5 gm/dl) |  |  |  |  |  |  |
| SUKARI KWENYE MKOJO (+) |  |  |  |  |  |  |
| UMRI WA MIMBA KWA WIKI |  |  |  |  |  |  |
| KIMO CHA MIMBA (BAADA YA WIKI 20 (cm) |  |  |  |  |  |  |
| MLALO WA MTOTO (KUANZIA WIKI YA 36) |  |  |  |  |  |  |
| KITANGULIZI (KUANZIA WIKI YA 36) |  |  |  |  |  |  |
| MTOTO ANACHEZA BAADA YA WIKI 20 (NDIYO/  HAPANA) |  |  |  |  |  |  |
| MAPIGO YA MOYO WA MTOTO (BAADA YA WIKI  20) YAPO (Y), HAKUNA (H) |  |  |  |  |  |  |
| KUVIMBA MIGUU/USO/MIKONO “Oedema” (++) |  |  |  |  |  |  |
| **DAWA ZA KINGA:** |  |  |  |  |  |  |
| Ferrous Sulphate (2 Kila siku) |  |  |  |  |  |  |
| *Folic acid (1 kila siku) |  |  |  |  |  |  |
| Mebendazole (500mg start) |  |  |  |  |  |  |
| Sulphadoxine/Pyrimethamine (SP) vidonge 3 baada  Ya wiki 20, rudia dozi hii baada ya wiki nne. (Baada  Ya kumeza SP) |  |  |  |  |  |  |

| 🗣 **INTERVIEWER: *“Thank you very much for the information and your time”.*** |
| --- |
